# From Inflammation to Infertility: How Oxidative Stress and Infections Disrupt Male Reproductive Health

**DOI:** 10.3390/metabo15040267

**Published:** 2025-04-11

**Authors:** Anastasios Potiris, Efthalia Moustakli, Eleni Trismpioti, Eirini Drakaki, Despoina Mavrogianni, Alkis Matsas, Athanasios Zikopoulos, Antonios Sfakianakis, Ioannis Tsakiridis, Themistoklis Dagklis, Athanasios Zachariou, Panagiotis Christopoulos, Ekaterini Domali, Peter Drakakis, Sofoklis Stavros

**Affiliations:** 1 Third Department of Obstetrics and Gynecology, University General Hospital “ATTIKON”, Medical School, National and Kapodistrian University of Athens, 124 62 Athens, Greece; thanzik92@gmail.com (A.Z.); pdrakakis@med.uoa.gr (P.D.); 2 Laboratory of Medical Genetics, Faculty of Medicine, School of Health Sciences, University of Ioannina, 451 10 Ioannina, Greece; thaleia.moustakli@gmail.com; 3 First Department of Obstetrics and Gynecology, Alexandra Hospital, Medical School, National and Kapodistrian University of Athens, 115 28 Athens, Greece; elenatrismpioti@gmail.com (E.T.); eirinidrak@med.uoa.gr (E.D.); dmavrogianni@med.uoa.gr (D.M.); kdomali@yahoo.fr (E.D.); 4 Laboratory of Experimental Surgery and Surgical Research “N.S. Christeas”, Medical School, National and Kapodistrian University of Athens, 115 27 Athens, Greece; amatsas@med.uoa.gr; 5 Homerton Fertility Centre, Homerton University Hospital, Homerton Row, Clapton, London E9 6SR, UK; antsfak@gmail.com; 6 Third Department of Obstetrics and Gynecology, General Hospital Ippokratio, Medical School, Aristotle University of Thessaloniki, 546 42 Thessaloniki, Greece; iotsakir@gmail.com (I.T.); tdagklis@gmail.com (T.D.); 7 Department of Urology, School of Medicine, Ioannina University, 45110 Ioannina, Greece; zahariou@otenet.gr; 8 Second Department of Obstetrics and Gynecology, Aretaieion University Hospital, Medical School, National and Kapodistrian University of Athens, 115 28 Athens, Greece

**Keywords:** oxidative stress, inflammation, microbiota, pro-inflammatory cytokines, spermatogenesis, antimicrobial therapy, reproductive health

## Abstract

Background/Objectives: Inflammation, infections, and oxidative stress (OS) all have an impact on male infertility, which is a complicated, multifaceted illness. OS affects motility and fertilization capability. It accomplishes this through damaging sperm DNA, oxidizing proteins, and triggering lipid peroxidation. These effects occur due to an imbalance between reactive oxygen species (ROS) and antioxidant defenses. Methods: This review aims to evaluate the impact of oxidative stress and inflammation on male infertility by assessing recent literature. Results: Pro-inflammatory cytokines, like TNF-α and IL-6, interfere with spermatogenesis and promote oxidative damage. Additionally, infections caused by pathogens like *Escherichia coli* and Chlamydia trachomatis alter the reproductive microenvironment, leading to sperm dysfunction and inflammation. Conclusions: Early detection and targeted treatment are essential due to the intricate interactions among these elements. Microbiota-modulating techniques, antimicrobial therapies, anti-inflammatory drugs, and antioxidants are therapeutic approaches that may help reduce oxidative damage and enhance male fertility.

## 1. Introduction

Aerobic metabolism depends on oxygen to support metabolic reactions that are vital to cellular activity. ROSs, which serve dual roles in biological systems, are also produced by these metabolic pathways. At physiological levels, ROSs support homeostasis and cellular signaling; however, excessive accumulation or depletion disrupts redox balance, leading to OS and cellular damage [1].

Aerobic organisms have developed complex antioxidant defense systems to lessen the cytotoxicity caused by ROSs. ROSs encompass a diverse range of molecules, broadly classified into free radicals and non-radical oxidants. Free radicals, distinguished by their unpaired electrons, include hydroxyl radicals (OH^−^), alkoxyl radicals, superoxide anions (O_2_^−^), and hydroperoxyl radicals. A similar study states that non-radical oxidants, such as hydrogen peroxide (H_2_O_2_) and ozone (O_3_), drive oxidative reactions despite lacking free radical properties [2].

Once believed to impair sperm function alone, ROSs are now recognized as vital regulators of male reproductive physiology. At controlled concentrations, ROSs play crucial roles in sperm motility, acrosomal response, capacitation, and gamete interaction. Tyrosine kinase activation, calcium influx, and ROS generation all contribute to capacitation—a process that enhances sperm function by increasing cyclic adenosine monophosphate (cAMP) levels. Experimental research indicates that sublethal ROS concentrations enhance oocyte penetration, acrosomal exocytosis, and capacitation. Key ROSs, including superoxide anions (O_2_^−^) and nitric oxide (NO•), facilitate these processes by regulating nuclear maturation and sperm membrane fluidity through controlled protein oxidation and lipid peroxidation [3].

ROSs further employ phosphorylation-dependent signaling to control sperm activity. The tyrosine phosphorylation of proteins, including ezrin, a cytoskeletal protein necessary for sperm activation, and the p105 component of nuclear factor-kappa B (NF-κB), is specifically mediated by sperm-derived H_2_O_2_. H2O_2_ mimics the action of bicarbonate, which is known to be an activator of adenylate cyclase in spermatozoa, by inhibiting tyrosine phosphatases and upregulating cAMP production simultaneously. This mechanism leads to tyrosine phosphorylation and sperm activation while also driving cAMP-dependent protein kinase A (PKA) signaling [4].

High ROS levels impair antioxidant defenses, leading to OS-induced protein oxidation, DNA breakage, and lipid peroxidation. These effects contribute to male infertility by reducing motility, disrupting acrosomal responses, and triggering apoptotic pathways, all of them compromising sperm integrity and function. Moreover, OS is closely linked to inflammation and infections, as both promote excessive ROS production and disrupt the reproductive environment [5].

Male infertility is primarily attributed to pathogenic infections, especially those caused by *Escherichia coli*, Chlamydia trachomatis, Ureaplasma urealyticum, and other sexually transmitted diseases. According to Shahini et al. (2022), these infections trigger inflammatory responses, resulting in increased levels of pro-inflammatory cytokines like interleukin-6 (IL-6) and tumor necrosis factor-alpha (TNF-α), which exacerbate oxidative damage [6]. Furthermore, bacterial toxins and lipopolysaccharides (LPS) hinder sperm function by compromising the blood–testis barrier, increasing ROS production, and disrupting mitochondrial activity. To enhance male fertility and alleviate reproductive dysfunction, targeted therapies that address the interplay between OS, infections, and inflammation are vital [7].

## 2. OS and Fertility Pathophysiological Mechanisms and Clinical Implications

OS results from an imbalance between antioxidant defenses and ROS production, with growing evidence linking this disruption to male infertility. Wang et al. (2019) report that excessive ROS accumulation leads to sperm dysfunction, lipid peroxidation, DNA fragmentation, and cell death [8]. However, regulated ROS levels are crucial for sperm capacitation, the acrosome reaction, and successful oocyte fertilization. Inflammation and infections worsen OS by activating the immune system, increasing leukocyte infiltration, and releasing pro-inflammatory cytokines that disrupt spermatogenesis and impair sperm quality.

### 2.1. The Role of Infection, Inflammation, and Leukocytes in OS

The main cause of male infertility is infections of the male reproductive system, which can be caused by viruses, fungi, and bacteria such as *Escherichia coli*, *Chlamydia trachomatis*, and *Ureaplasma urealyticum*. ROSs are overproduced in several illnesses. Pathogenic bacteria not only damage sperm cells directly by secreting toxins, but they also indirectly injure them by inducing inflammatory processes. For example, the activation of toll-like receptor (TLR)-mediated signaling by lipopolysaccharides (LPSs) from Gram-negative bacteria like E. coli results in the production of pro-inflammatory cytokines (TNF-α, IL-6, and IL-8) and the activation of nuclear factor kappa B (NF-κB). These inflammatory mediators increase ROS levels, impair sperm motility, and harm the integrity of the sperm membrane and DNA [9].

ROSs are also primarily produced by leukocytes in seminal plasma. The production of superoxide anions (O_2_^−^), hydrogen peroxide (H_2_O_2_), and hypochlorous acid (HOCl) by neutrophils and macrophages that are drawn to the site of infection or inflammation exacerbates OS. According to studies, leukocytospermia, a disorder that severely reduces sperm function, is present in 10–20% of infertile males [10]. Leukocyte concentrations greater than 10^6^ cells/mL of semen are considered leukocytospermia by the World Health Organization (WHO). The release of ROSs, chemotactic factors, and proteases by activated macrophages triggers inflammatory signaling pathways and raises ROS levels even further. The balance between antioxidant defenses and ROS levels is upset by this OS, which activates chemokines including CXCL5, CXCL8, IL-6, and IL-8. This ultimately affects sperm function and contributes to male infertility. In infected individuals, high levels of IL-1, IL-18, and TNF-α can lead to blood-testis barrier disruption, testicular microenvironment alterations, and increased DNA damage [7,8].

### 2.2. Mitochondrial Dysfunction as a Key Mediator of OS

Mitochondria are the main generators of ROS in spermatozoa and are mainly susceptible to OS. ROSs promote two primary types of damage to sperm cells: they disrupt membrane integrity and induce DNA fragmentation. Lipid peroxidation impairs motility by decreasing membrane fluidity. Oxidative DNA damage includes single- and double-strand breaks, base modifications, and cross-linking, which damage genetic integrity and increase the risk of mutations that could interfere with conception [11].

OS is linked to telomere shortening, cellular aging, and potentially reduced embryo viability. Malondialdehyde (MDA) is a highly reactive carcinogenic aldehyde and a notable byproduct of lipid peroxidation. MDA promotes ROS production by altering the structure of mitochondrial membranes [12]. As a result, mitochondria create a harmful cycle, acting as both a source and a target of OS. Since mitochondrial DNA (mtDNA) lacks protective histones and has limited repair capabilities, it is more susceptible to oxidative damage than nuclear DNA. Extensive mitochondrial damage often prevents spermatozoa from undergoing effective apoptosis, leading to sperm with compromised DNA integrity [13,14].

Beyond direct cellular damage, OS affects sperm bioenergetics. Mitochondrial dysfunction reduces ATP production, impairing germ cell differentiation and disrupting spermatogenesis. Consequently, sperm maturation is compromised, leading to decreased fertility potential [15].

### 2.3. Lipid Peroxidation and Sperm Function

Oxidative degradation compromises the structural integrity and membrane fluidity of PUFAs. Higher levels of MDA further impair mitochondrial function, with this adverse impact on sperm motility, energy levels, and fertilization ability. Membrane damage further interferes with sperm–oocyte union, complicating fertilization [8]. Reduced membrane fluidity prevents sperm from reaching the oocyte through the acrosome response. Bacterial infections that produce toxins, such as hemolysins and lipopolysaccharides (LPS), disrupt the sperm membrane and promote phospholipid oxidation, aggravating OS [16]. Therefore, the membrane becomes weaker, making the sperm more vulnerable to ROSs and compromising fertility. The complex interaction between lipid peroxidation, bacterial toxins, and OS underscores the delicate balance required to preserve sperm health and fertility [17].

### 2.4. OS, Telomere Damage, and Apoptosis in Sperm Health

Maintaining genomic stability mainly relies on the repetitive, guanine-rich telomeres at the ends of chromosomes. Telomeres are particularly vulnerable to OS because guanine has a low oxidation potential. This can lead to carcinogenic damage, including the formation of 8-hydroxy-deoxyguanosine (8-OHdG). Oxidative damage to telomeres can contribute to male infertility by accelerating cellular aging and apoptosis in germ cells, ultimately reducing sperm count [18].

To maintain sperm quality, defective germ cells are eliminated through apoptosis, also known as programmed cell death, during spermatogenesis. Furthermore, it maintains cellular homeostasis and promotes tissue growth. This process is controlled by caspases, a group of proteolytic enzymes involved in cytoskeletal breakdown, DNA fragmentation, and chromatin condensation. Any dysregulation in apoptosis, whether through excessive or inhibited activity, can affect sperm function and production [19].

During spermatogenesis, apoptosis is essential for preserving the balance of germ cells, as a considerable percentage of spermatogonia experience programmed cell death. Mutations or changes in apoptotic regulators such as BAX and BCL-2 can reduce sperm production and fertility by affecting germ-cell survival and altering the Sertoli cell-to-germ-cell ratio. Furthermore, apoptosis ensures that only functionally competent gametes mature by removing spermatozoa with genetic abnormalities [20].

Substances such as alcohol, heavy metals, toxins, and chemotherapy drugs like cisplatin can induce germ cell death. Increased ROS levels have also been linked to prolonged exposure to electromagnetic radiation from mobile devices and Wi-Fi networks, which may trigger apoptosis in spermatozoa [21].

OS plays a major role in triggering apoptosis in male germ cells, leading to DNA damage and chromatin degradation due to elevated ROS levels and inadequate antioxidant defenses. Subfertile men frequently exhibit increased apoptotic activity, including caspase activation and greater DNA damage, further compromising sperm function [22]. Atypical chromatin architecture promotes sperm apoptosis, which diminishes the quantity of viable, motile sperm and reproductive potential. Excessive apoptosis can significantly reduce the quantity and quality of sperm, which can lead to male infertility. Moreover, reduced motility and structural abnormalities, both essential for successful fertilization, are frequently observed in spermatozoa from infertile men [23].

Excessive ROS production stimulates TNF receptor interactions, increasing nuclear factor kappa B (NF-κB) activity and pro-inflammatory cytokine levels. However, this process can inhibit apoptosis, allowing genetically defective sperm to persist [24]. Mitochondrial dysfunction further reduces sperm viability by promoting ROS accumulation and decreasing ATP production. Although mitochondrial abnormalities and NF-κB activation may allow defective sperm to remain in the ejaculate and prevent complete cell death, some sperm still undergo apoptosis. Therefore, preserving the integrity of both nuclear and mitochondrial DNA is necessary to preserve male fertility [25].

### 2.5. How Do Free Radicals Target the Sperm Ion Channels and After Sperm Function?

Sperm cells depend on the precise regulation of ion channels to maintain motility, capacitation, and fertilization potential. Free radicals, particularly ROSs, play a dual role in sperm development. At physiological levels, ROSs are essential for sperm capacitation and acrosome reactions. However, excessive ROS generation leads to OS, which damages sperm ion channels and impairs sperm function. Cation Channel of Sperm (CatSper), a calcium ion channel, plays a crucial role in sperm hyperactivation. Free radicals cause oxidative changes in CatSper proteins, affecting their function, lowering sperm motility, and limiting fertilization capacity. Voltage-Gated Proton Channel (Hv1) regulates intracellular pH by extruding protons, a crucial process for sperm capacitation. ROSs inhibit Hv1 function, resulting in acidification of the sperm cytoplasm and decreased motility. Potassium Channel of Sperm (KSper) facilitates potassium efflux, which is crucial for membrane hyperpolarization during capacitation. OS alters KSper function, leading to decreased sperm viability and disrupted membrane potential [26].

Free radicals cause harm via a variety of processes. Lipid peroxidation affects polyunsaturated fatty acids in sperm membranes, leading to structural changes in ion channels. Protein oxidation modifies amino acid residues in ion channel proteins, causing conformational alterations that impair their function. Mitochondrial dysfunction caused by ROS-induced damage leads to ATP depletion, affecting energy-dependent ion channel activity and reducing sperm motility. These disruptions cause reduced motility, impaired capacitation, and DNA damage. Dysfunctional ion channels hinder calcium influx and pH regulation, both essential for sperm movement. OS inhibits the membrane hyperpolarization necessary for capacitation, preventing sperm from achieving their full fertilization potential. Exposure to ROSs also leads to sperm DNA fragmentation, significantly reducing reproductive potential [27].

**Figure 1:**
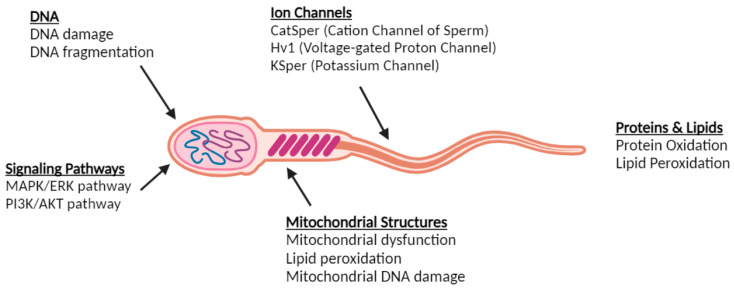
Targets of free radicals in sperm cells. Free radicals can induce oxidative damage in various components of sperm, including ion channels (Cation Channel of Sperm, Voltage-Gated Proton Channel, Potassium Channel), DNA (DNA damage, DNA fragmentation), and mitochondrial structures (mitochondrial dysfunction, lipid peroxidation, mitochondrial DNA damage). Additionally, oxidative stress affects proteins and lipids (protein oxidation, lipid peroxidation) and disrupts key signaling pathways (MAPK/ERK and PI3K/AKT pathways), ultimately impairing sperm function and fertility.

Antioxidant therapy using vitamins C, E, and glutathione may offer a preventive strategy by reducing oxidative damage to sperm ion channels. Enzymatic defense systems such as superoxide dismutase (SOD) and catalase are critical for neutralizing excess ROSs and preserving sperm integrity. Lifestyle changes, such as limiting exposure to environmental toxins, smoking, and eating unhealthy foods, can reduce OS and protect sperm function. Understanding how free radicals target sperm ion channels sheds light on male infertility causes and emphasizes the importance of focused therapy approaches to protect sperm function from oxidative damage [28]. Figure 1 illustrates the multiple ways in which free radicals can induce oxidative damage in various components of sperm.

### 2.6. Downstream Signaling Mechanisms of Free Radicals in the Regulation of Sperm Function

Free radicals, particularly ROSs, affect sperm function through various intracellular signaling pathways. Normal ROS levels are vital for physiological processes like capacitation, hyperactivation, and the acrosome reaction. However, excessive ROS production results in OS, which impairs sperm function [29].

The mitogen-activated protein kinase (MAPK) pathway is an important downstream signaling pathway that is influenced by free radicals. ROSs stimulate MAPKs, including ERK1/2, JNK, and p38, which control phosphorylation events required for sperm motility and capacitation. However, severe ROS activation of the MAPK pathway can cause apoptosis and decreased sperm viability. Similarly, ROSs influence the phosphoinositide 3-kinase (PI3K)/Akt signaling pathway, with moderate activation supporting sperm survival and motility and high OS inhibiting Akt phosphorylation, resulting in mitochondrial malfunction and apoptosis [30].

ROSs also modulate calcium signaling, a process crucial for sperm function. OS disrupts calcium homeostasis by altering channels like CatSper, resulting in abnormal sperm hyperactivation. ROSs also have an effect on the cyclic adenosine monophosphate (cAMP)/protein kinase A (PKA) pathway, which regulates sperm motility and capacitation by altering protein phosphorylation. Excess ROSs can disrupt this mechanism, lowering sperm fertilization potential [31].

Furthermore, OS impairs mitochondrial function, creating more ROSs and causing a vicious cycle of damage. This mitochondrial malfunction lowers ATP generation, which directly affects sperm motility. ROSs target the NF-κB signaling system, which regulates inflammation and can cause sperm DNA breakage and impaired fertility [32].

Understanding these downstream signaling mechanisms provides insights into the balance between beneficial and harmful effects of ROSs in sperm function. Targeting these pathways through antioxidants and therapeutic interventions may help mitigate oxidative damage and improve male fertility outcomes [33].

## 3. Causes of OS in Male Reproductive Health

Male infertility is significantly influenced by OS, which is affected by lifestyle decisions, health issues, and environmental exposures. Although the impact of OS on reproductive health is well-established, particularly in conditions such as varicocele, more research is required to comprehend the effects of emerging causes, like hyperhomocysteinemia [34].

According to research, an estimated 37 million men suffer from idiopathic male infertility [35]. High levels of ROSs and compromised antioxidant defenses are common, even in men with normal sperm motility and morphology. Maintaining oxidative balance is essential for male reproductive health, as emphasized by previous studies [36,37].

Significantly contributing to sperm oxidative damage are chronic disorders such as hemoglobinopathies, diabetes, thyroid dysfunction, and hyperhomocysteinemia (associated with folate insufficiency). For instance, elevated glucose metabolism in diabetes leads to hyperglycemia, which triggers excessive ROS generation. This disruption of mitochondrial activity and increased sperm DNA fragmentation impede the possibility of fertilization [38]. Although further research is needed, hyperhomocysteinemia, which is associated with folate deficiency and altered methylation processes, may also elevate OS in sperm cells [39,40].

OS is exacerbated by unhealthy lifestyle choices. Alcohol consumption impairs mitochondrial function and leads to DNA fragmentation, while smoking elevates leukocyte concentration and ROS levels in sperm [37]. Sperm quality is further reduced by obesity, poor diet, and lack of physical activity, all of which contribute to systemic inflammation and mitochondrial dysfunction. These lifestyle factors make sperm cells more susceptible to oxidative damage by impairing the antioxidant defense system [41,42].

Environmental pollutants also play a critical role in sperm oxidative damage. Industrial chemicals, pesticides, and heavy metals generate free radicals that interfere with oxidative phosphorylation and mitochondrial function. According to Pizzol et al. (2021), spermatogenesis is significantly impaired by pesticides and heavy metals, such as lead and cadmium, which elevate ROS levels in the testes [43]. Varicocele, characterized by enlarged testicular veins, exacerbates OS by reducing testicular blood flow, increasing ROS levels, and impairing fertility. According to Chung et al. (2011), cryptorchidism can also cause long-term oxidative damage to sperm quality, even after surgical repair [44].

**Figure 2:**
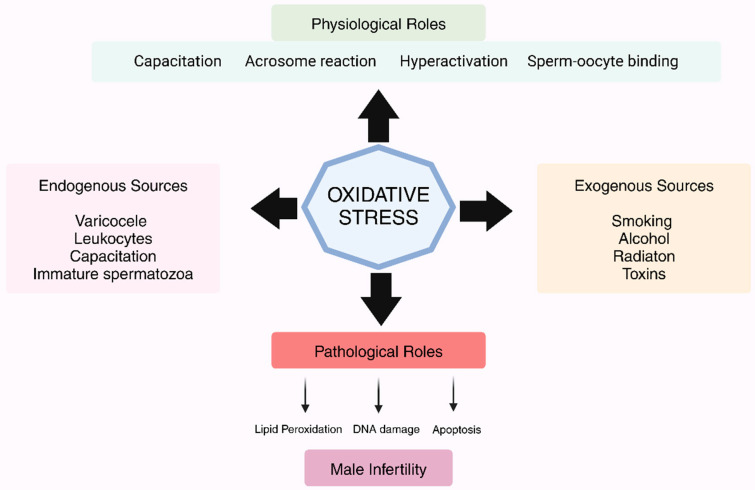
The causes and the impact of OS in male infertility.

Viral and bacterial infections have a significant impact on oxidative damage to sperm. Pathogens such as *Escherichia coli* and Chlamydia trachomatis, along with viruses like herpes simplex virus (HSV-1), trigger inflammatory responses that elevate ROS levels and impair sperm function [9]. Pro-inflammatory cytokines, such as TNF-α, IL-6, and IL-8, are released during inflammation caused by infection. This results in reduced sperm motility, damage to the sperm membrane, and an increase in ROS production [37,45]. Elevated ROS levels in fatigued individuals further compromise the integrity of sperm DNA, reducing reproductive capacity [46]. Figure 2 summarizes the causes of OS and their impact on male infertility.

### Exogenous Parameters and Their Role in Free Radical Generation and Sperm Function

Various external variables contribute to the excessive production of free radicals, which causes OS and impairs sperm function. Environmental contaminants, microbial infections, and heavy metals are major contributors to male infertility [47].

Air pollution, insecticides, and endocrine-disrupting chemicals (EDCs) all generate ROSs through metabolic activation and inflammatory reactions. Pollutants, including polycyclic aromatic hydrocarbons (PAHs) and bisphenol A (BPA), disturb sperm cells’ redox balance, causing lipid peroxidation, DNA damage, and mitochondrial malfunction, reducing motility and fertilization potential. Another important external factor that causes ROS production is microbial infections in the male reproductive tract. Pathogenic bacteria and viruses elicit immunological responses, including neutrophil activation and the production of inflammatory mediators. This immunological response enhances OS, which damages sperm membranes, causes DNA breakage, and reduces sperm viability. Microbial dysbiosis also affects the microbiota in the reproductive tract, worsening OS and inflammation [48].

Heavy metals, such as lead (Pb), cadmium (Cd), and mercury (Hg), also significantly contribute OS in sperm cells. These hazardous metals inhibit antioxidant enzyme activity, resulting in excessive ROS buildup. Cadmium, for example, inhibits mitochondrial activity and causes apoptosis in sperm cells, whereas lead exposure is associated with lower sperm motility and increased morphological abnormalities. Mercury compounds cause toxicity by attaching to thiol groups in proteins, resulting in oxidative damage and reduced sperm function [49].

The interplay between these exogenous parameters and OS creates a vicious cycle, ultimately reducing male fertility. Strategies to mitigate their impact include lifestyle modifications, dietary antioxidant supplementation, and reducing exposure to environmental and occupational toxins. Understanding these relationships is critical for developing protective interventions aimed at preserving sperm health and improving reproductive outcomes [50].

## 4. Biomarkers and Diagnosis

OS markers are essential for assessing male reproductive health by evaluating ROS levels, antioxidant capacity, and oxidative damage to critical biomolecules like proteins, DNA, and lipids [51]. Key markers include MDA, which signifies lipid peroxidation in seminal plasma, and glutathione (GSH), an indicator of the antioxidant capacity of sperm and seminal plasma. Increased MDA or decreased GSH levels indicate oxidative damage to the sperm membrane, which may impair motility and fertility. Combined with conventional semen analysis, these biomarkers provide a more thorough assessment of male fertility [52].

Table 1 provides a detailed summary of key OS indicators, their assessment methods, and clinical significance. Increased OS is associated with asthenozoospermia, teratozoospermia, elevated semen density, and compromised sperm quality. Critical insights into sperm quality can be gained from chromatin condensation and DNA fragmentation. Employing OS markers in traditional semen analysis enhances the evaluation of male infertility and promotes more individualized treatment approaches.

## 5. Antioxidants and Their Role in Counteracting OS

OS is commonly associated with male infertility because of its crucial involvement in sperm cell destruction. Antioxidants are crucial for reducing this stress, as they neutralize ROSs. However, antioxidant supplements should be used with caution. Uncontrolled use of antioxidants can disrupt the delicate balance required for optimal sperm function [51].

According to Zini and Al-Hathal (2011), antioxidants may increase sperm motility, without impacting sperm DNA integrity, which is crucial for the effectiveness of assisted reproductive technology (ART) [53]. Vitamins C and E, selenium, zinc, and glutathione are examples of antioxidant therapies that may neutralize ROSs and improve fertilization rates [54]. Semen contains both enzymatic and non-enzymatic antioxidants that protect sperm. Zinc and selenium deficiencies are associated with reduced fertilization capacity and sperm DNA damage. These nutrients are essential for defending against OS [55].

### 5.1. Enzymatic Antioxidant Defense Mechanisms in the Body

According to Jomova et al. (2024), the body uses several antioxidant systems, such as glutathione reductase, glutathione peroxidase, catalase, and superoxide dismutase (SOD), to fight ROS and shield sperm from OS [56]. These enzymes neutralize various forms of ROSs, thereby protecting sperm from oxidative damage. SOD catalyzes the conversion of superoxide (O_2_^−^) into molecular oxygen (O_2_) and hydrogen peroxide (H_2_O_2_) through the following reaction: 2O_2_^−^ + 2H⁺ → H_2_O_2_ + O_2_. The three forms of SOD—cytosolic (SOD1), mitochondrial (SOD2), and extracellular (SOD3)—play a crucial role in sperm health. Mitochondrial SOD2 is especially important because mitochondria generate a significant amount of ROS during oxidative phosphorylation. The Leydig and Sertoli cells in the testes also generate SOD [57].

Sperm from men with oligoasthenoazoospermia show significantly lower SOD activity compared to those without the condition [58]. This leads to greater oxidative damage in the sperm mitochondria, which in turn reduces motility and DNA integrity. Catalase, found mainly in the cytoplasm and peroxisomes, helps neutralize hydrogen peroxide (H_2_O_2_) by converting it into water and oxygen through the reaction: 2H_2_O_2_ → 2H_2_O + O_2_ [59].

Catalase activity is critical for sperm health since H_2_O_2_ can oxidize lipid membranes, leading to lipid peroxidation. According to Rubio-Riquelme et al. (2020), this reduces membrane fluidity, thereby decreasing motility and fertilization potential [60].

Glutathione peroxidase uses reduced glutathione (GSH) to neutralize H_2_O_2_, converting it into water and oxidized glutathione (GSSG) through the reaction: H_2_O_2_ + 2 GSH → 2H_2_O + GSSG. Glutathione reductase (GR) regenerates GSH from GSSG using NADPH, as shown in the reaction: GSSG + NADPH + H⁺ → 2 GSH + NADP + GSSG. Sperm protection against OS is largely dependent on the glutathione system. Low glutathione activity is associated with increased DNA damage and lipid peroxidation, which may reduce fertilization potential and increase the risk of miscarriages [61,62].

### 5.2. Non-Enzymatic Antioxidant Defense of the Body

The body utilizes enzymatic and non-enzymatic antioxidants, such as glutathione (GSH), melatonin, ferritin, and albumin, to combat OS. GSH, the primary natural antioxidant, is vital for maintaining cellular homeostasis. It is synthesized in cells to regulate redox potential and protect biomolecules from oxidative damage. The sulfhydryl group (-SH) in reduced GSH reacts with free radicals, converting them into less reactive molecules. GSH exists in two forms: the active reduced form (GSH) and the oxidized form (GSSG), which is produced when ROS binds. Under normal conditions, cells maintain a high GSH/GSSG ratio, indicating a strong antioxidant environment [63].

A decrease in reduced glutathione and an increase in its oxidized form signals OS and cellular dysfunction. To prevent OS, GSH plays a role in detoxification, immune response regulation, toxin metabolism, and maintaining protein integrity. In terms of male fertility, it protects sperm membranes and DNA from oxidative damage. A deficiency in GSH is linked to reduced sperm motility and increased DNA fragmentation, both of which impair fertilization ability. GSH supplementation has been demonstrated to enhance sperm quality in infertile males with varicocele or urinary tract inflammation [64,65].

Melatonin functions both as an antioxidant and a hormone, produced by various organs in the body. Reducing heat-induced OS can improve sperm motility, lower mitochondrial ROSs, stabilize mitochondrial membrane potential, decrease lipid peroxidation, preserve DNA integrity, and reduce sperm cell death [66].

Ferritin and myoglobin protect sperm from oxidative damage by neutralizing pro-oxidant metal ions. Ferritin, which safeguards testicular tissues and stores iron necessary for sperm motility and function, along with non-heme ferritin, helps regulate sperm pH and viscosity, thus influencing spermatogenesis. Excess iron is bound by mitochondrial ferritin (MtF), expressed in sperm and Leydig cells, which halts the Fenton reaction and protects mitochondria from OS. High MtF levels reduce cytoplasmic ferritin and increase transferrin receptor expression, ensuring sufficient iron for spermatogenesis [67]. Albumin, found to be lower in infertile men, likely functions as an antioxidant by providing thiol groups for its activity [68].

### 5.3. Dietary and Supplementary Antioxidants

Ascorbic acid (vitamin C), a vital cofactor in hydroxylation processes, is a water-soluble antioxidant. It is found in high concentrations in seminal plasma, where it helps protect against DNA damage. In contrast, vitamin E, a fat-soluble antioxidant, safeguards cell membranes by neutralizing free radicals. Furthermore, it has been demonstrated that vitamin E prevents infertile males from producing ROS [69].

Research by Greco et al. (2005) found that daily supplementation with 1 g of vitamin C and 1 g of vitamin E for two months reduced DNA damage [70]. However, important sperm characteristics like concentration and motility were not considerably impacted by this supplementation. The human body metabolizes the nutrient L-carnitine. Because of its constant secretion in the epididymis, it is present in great concentrations there, with levels more than 2000 times higher than in serum [71]. Studies suggest a positive correlation between sperm motility and elevated levels of L-carnitine in the epididymis and L-acetyl carnitine (LAC) in sperm. Another study revealed that sperm motility was enhanced after six months of supplementing with 2 g/day of L-carnitine and 1 g/day of LAC [72]. Further adding to the conflicting data about carnitine’s function as an antioxidant in sperm is the fact that other studies did not discover any meaningful association between this therapy and sperm characteristics [73].

Coenzyme Q10 (CoQ10), also known as ubiquinoneis, is a vital antioxidant involved in cellular energy production. As a component of the electron transport chain, CoQ10 facilitates the production of ATP, the primary energy carrier in cells. The effects of CoQ10 on infertile males were studied by Balercia et al. (2009), who found that sperm motility and CoQ10 concentrations increased after six months of treatment [74]. Notably, the treatment group experienced six pregnancies, while the placebo group had only three.

CoQ10 supplementation has been shown in several studies to enhance sperm traits as motility, concentration, morphology, and antioxidant capability [75]. A meta-analysis, however, indicated that while CoQ10 supplementation improved sperm parameters, it had no discernible effect on infertile men’s chances of causing pregnancy [76].

Zinc is a common mineral in the body and has been shown to protect sperm from oxidative damage, potentially restoring impaired sperm function. Studies indicate that consistent zinc supplementation improves sperm quality and helps normalize oxidized thiol levels. Zinc is also essential for sperm maturation and testicular development [77,78]. Low zinc levels in sperm are linked to a reduced ability to fertilize. Although some studies suggest the opposite effect, it has been demonstrated that supplements containing zinc and folic acid can increase sperm concentration [77]. Selenium, an essential trace element, plays a crucial role in sperm formation and testosterone biosynthesis. Proteins that bind selenium help maintain sperm integrity. Selenium supplementation increased sperm count, concentration, and overall quality in infertile males, according to results from clinical trials conducted in Iran and Tunisia [79].

The amino acid L-cysteine is the natural source of N-acetylcysteine (NAC), a substance that acts as a precursor to glutathione peroxidase. It has been shown that co-administration of NAC and selenium in infertile men increases testosterone levels, reduces follicle-stimulating hormone (FSH), and improves sperm quality. Although the results of various studies have varied, these antioxidants likely benefit sperm [80,81].

## 6. Infections of the Genital Tract

Bacterial, viral, or protozoan infections of the male genital tract can severely affect fertility. Common sexually transmitted infections (STIs) like syphilis, chlamydia, and gonorrhea trigger immune and inflammatory responses, which are critical for reproductive health. These infections often lead to OS through the production of ROSs, which disrupt sperm function [82].

Certain conditions can severely affect reproduction when they become active, even if they are asymptomatic during their latent phase. For example, Neisseria gonorrhoeae, the bacterium responsible for gonorrhea, can cause urethritis, prostatitis, and epididymitis, which impair the testicles or block the spermatic canal, leading to infertility [83]. Gonorrhea compromises sperm function by increasing ROS levels, damaging sperm cells, and promoting leukocyte migration. *Escherichia coli*, a common cause of urinary tract and accessory gland infections, also affects sperm motility, acrosomal response, and fertilization capacity [84]. Notably, E. coli destroys sperm membranes and increases the generation of ROSs, according to Gimenes et al. [85]. Moreover, Ureaplasma species, such as Ureaplasma urealyticum, interfere with spermatogenesis and change the seminal fluid, which reduces its protective function and increases the formation of ROSs, hence contributing to infertility [86].

Similarly, Mycoplasma hominis affects sperm motility and quality by inducing urogenital tract inflammation and promoting bacterial adhesion to sperm cells, resulting in cellular alterations and oxidative damage [9]. Chlamydia trachomatis, the most common sexually transmitted infection worldwide, damages male germ cells and Sertoli cells, impairs sperm motility and integrity, and can lead to infertility. Due to their silent nature, chronic infections sometimes go undetected, raising the possibility of long-term fertility issues [87].

### Neutrophil Extracellular Traps (NETs) and Their Role in Infection and Sperm Function

Neutrophil extracellular traps (NETs) are web-like structures composed of decondensed chromatin, histones, and antimicrobial proteins formed by neutrophils during the innate immune response. NETs are typically produced in response to illnesses, and they catch and kill invading microbes, preventing them from spreading. While this mechanism is crucial for immune defense, excessive or dysregulated NET production can result in inflammation and tissue damage [88].

In the context of male reproductive health, NETs have been linked to infections of the male reproductive tract. Bacterial and viral infections cause the production of NETs in the seminal plasma and reproductive organs. While NETs effectively trap infections, their components, such as myeloperoxidase, elastase, and histones, cause OS, impairing sperm function. The presence of NETs in the seminal fluid has been associated with increased ROS levels, which cause sperm DNA breakage, lipid peroxidation, and decreased motility [89].

Furthermore, NETs contribute to immune-mediated infertility by inducing chronic inflammation in the male reproductive system. Excessive NET formation may block and harm sperm cells, lowering their viability and fertility potential. Inflammatory cytokines produced by NETs exacerbate OS, creating an unfavorable environment for sperm function [90].

Understanding the role of NETs in infection-induced male infertility is crucial for developing targeted treatments. Strategies targeted at preventing NET development or neutralizing their damaging components, such as antioxidant therapy or anti-inflammatory drugs, may assist in minimizing NETs’ deleterious impact on sperm health and reproductive outcomes [91].

## 7. Microbiota on Male Reproductive System and OS

Several microbes in the male reproductive system play a crucial role in maintaining urogenital health. Infections and inflammatory responses that disrupt this balance can negatively affect reproductive function. Sexually transmitted infections, including Chlamydia trachomatis, Mycoplasma species, Trichomonas vaginalis, and Neisseria gonorrhoeae, typically enter the body through the urethra [7].

Such microbes cause immune cells to emit ROSs and pro-inflammatory cytokines as part of the host’s defense response. While the immune system relies on controlled ROS production, excessive ROS can damage the urethral epithelium, lead to chronic infections, and interfere with sperm transport. Chronic urethritis from these bacteria may allow infections to spread to deeper reproductive organs, worsening OS damage [92].

Uropathogens such as *Pseudomonas aeruginosa*, *Escherichia coli*, *Klebsiella* spp., *Proteus* spp., and *Enterococcus* spp. commonly invade the prostate, which contributes essential components to seminal fluid. In bacterial prostatitis, inflammation, OS, and dysfunction of prostatic secretions can occur. The prostate’s high concentration of polyunsaturated fatty acids makes it especially vulnerable to ROS-induced damage [93].

Studies indicate that OS in prostatitis alters the composition of prostatic fluid, reducing levels of antioxidant enzymes and zinc, both of which are vital for sperm protection. Overproduction of ROSs in the prostate also leads to sperm DNA fragmentation, which hinders the development of embryos and the capacity to fertilize them. Prostatic fibrosis has been linked to persistent OS in chronic prostatitis, which further impairs the gland’s functionality and decreases male fertility [94].

Infections such as Neisseria gonorrhoeae, Chlamydia trachomatis, and *Escherichia coli* can affect the epididymis, a key organ for sperm maturation, storage, and transport, leading to tissue damage, inflammation, and OS. Antioxidant enzymes like SOD and GPx are secreted by the epididymal epithelium to shield sperm from oxidative damage while in transit. However, infection-induced inflammation can weaken these defenses, leading to increased OS and sperm failure. Chronic epididymal infections are also associated with elevated levels of NO and peroxynitrite, which exacerbate lipid peroxidation and negatively affect sperm motility, viability, and oocyte fertilization [95].

Given its crucial role in spermatogenesis, which depends on a finely balanced redox environment, the testis is highly susceptible to OS. Orchitis, an inflammatory condition that impairs testicular function, can be caused by bacterial pathogens such as Neisseria gonorrhoeae, Chlamydia trachomatis, *Escherichia coli*, *Klebsiella* spp., *Pseudomonas aeruginosa*, *Staphylococcus* spp., and *Streptococcus* spp., as well as viral infections like mumps and Coxsackie B viruses [96].

Orchitis-induced OS directly damages spermatogenic and Sertoli cells, which are crucial for sperm formation, by increasing ROS production in the seminiferous tubules. ROS-mediated lipid peroxidation affects the integrity of sperm membranes, reducing their ability to survive in the female reproductive system. Chronic testicular infections worsen fertility issues by reducing testosterone production in Leydig cells [97].

**Figure 3:**
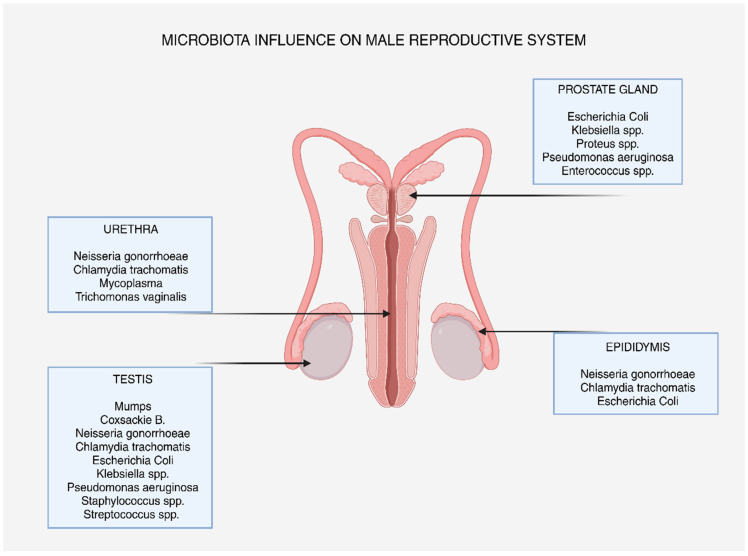
The influence of microbiota on male reproductive system.

To reduce oxidative damage and manage infections, probiotics, antioxidant therapy, and targeted anti-inflammatory treatments should be considered. Early detection and treatment of infections, coupled with strategies to restore microbiota balance, are crucial for maintaining male reproductive health [82]. Figure 3 depicts the most common pathogens in each part of the male genital tract.

## 8. The Role of Inflammation and OS

In response to harmful stimuli such as pathogens, chemicals, or cell damage, the body utilizes inflammation as a defense mechanism. While the primary purpose of inflammation is to protect the host, it might further lead to OS, disrupting cellular homeostasis. According to Azenabor, Ekun, and Akinloye, inflammation is a major factor in several male reproductive system disorders that can impair fertility. These include varicocele, testicular torsion, urethritis, infections, obstruction of the seminal tract, and epididymitis [98].

Acute inflammation is characterized by enhanced blood flow, increased capillary permeability, and the migration of immune cells to the site of injury. If acute inflammation is not addressed, it can develop into chronic inflammation, marked by the activation of macrophages and lymphocytes, along with the release of pro-inflammatory cytokines. Both chronic inflammation and the use of immunosuppressive drugs can greatly impair sperm quality by disrupting the function of accessory glands, obstructing sperm transport, and directly affecting spermatogenesis [90].

## 9. Impact of Inflammation and Systemic Factors on Male Fertility

The inflammatory response releases various cytokines, chemokines, and adhesion molecules based on the pathogen and immune response. PAMPs and DAMPs activate Toll-like receptors (TLRs), triggering multiple signaling pathways. This results in the release of pro-inflammatory cytokines like TNF-α, IL-1β, and IL-6, as well as chemokines like CXCL8 and CXCL10. These factors can negatively affect male reproductive health by targeting Sertoli cells, which are crucial for spermatogenesis, and Leydig cells, which are involved in testosterone production [99,100,101,102,103]. Specifically, TNF-α and TGF-β suppress the genes responsible for testosterone production during steroidogenesis by activating NF-kB subunits. Furthermore, TNF-α activates caspase-3, causing Sertoli cells to release CXCL10, which may trigger germ cell death. Chemokines induced by inflammation, such as CXCL10 and CCL2, recruit leukocytes to the infection site, thereby further disrupting spermatogenesis [104].

Systemic inflammation, often associated with conditions including obesity, may alter the effects of infections and further impair male fertility. Obesity disrupts the hypothalamic–pituitary–gonadal (HPG) axis, resulting in increased apoptosis and a decline in sperm motility, morphology, count, and DNA integrity. Obese males with excess white adipose tissue had higher estrogen levels, disturbed hormone balance, and worsened testicular dysfunction. Additionally, the inflammation caused by obesity, along with the cytokines it releases, can further aggravate reproductive health issues by reducing testosterone levels and increasing ROS, potentially contributing to erectile dysfunction [103,104]. Moreover, viral infections like SARS-CoV-2 also trigger inflammatory responses, particularly raising cytokine levels like IL-6, TNF-α, and IL-1β, which disrupt Sertoli cell function and reduce sperm count. This illustrates the profound influence of systemic inflammation, both from infections and conditions like obesity, on male fertility [105].

## 10. Discussion

Infections, inflammation, and OS are major contributors to male infertility, affecting approximately 15% of couples worldwide. The imbalance between the body’s antioxidant defenses and ROS leads to oxidative damage, which sperm cells are inefficient at preventing. This imbalance results in protein oxidation, lipid peroxidation, and DNA damage, negatively impacting sperm motility, viability, morphology, and fertilization potential. Severe OS is also linked to an increased risk of pregnancy loss and complications in fetal development [51,106].

Male infertility is often driven by microbial infections, which induce both inflammation and OS, further compromising reproductive function. Immune responses triggered by infections with pathogens such as *Mycoplasma*, *Ureaplasma*, *Neisseria gonorrhoeae*, *Chlamydia trachomatis*, and others generate ROSs and pro-inflammatory cytokines that damage sperm and hinder spermatogenesis [107]. Infections in the testis, prostate, urethra, and epididymis exacerbate OS-induced sperm damage through localized and systemic inflammation. Epididymal and testicular infections interfere with sperm maturation and spermatogenesis, while prostatitis, often caused by *Escherichia coli* and other bacteria, diminishes the antioxidant defenses necessary for sperm protection [7].

Therapies aimed at reducing oxidative damage and treating underlying infections should be prioritized in the management of infection-induced OS. Antioxidant treatments, such as N-acetylcysteine (NAC), CoQ10, and vitamins C and E, show promise in improving sperm function in affected individuals. Probiotic supplementation may also help reduce inflammation and restore microbiota balance. Early detection of infections and appropriate antimicrobial treatment can prevent long-term reproductive issues; however, careful antibiotic use is essential to avoid disrupting beneficial microbiota and exacerbating OS [108,109].

Emerging approaches to treat OS in male infertility, such as redox-modulating medications and anti-inflammatory drugs, may offer promising options. To further reduce OS and inflammation, lifestyle modifications, including maintaining a healthy body weight, reducing alcohol consumption, and quitting smoking, are critical. Despite the promise of antioxidant supplements and other treatments, clinical trials have produced mixed results, highlighting the need for a systematic approach to managing infection-induced male infertility [51].

Given the pivotal role of OS in male fertility, it is essential to prioritize infection control, OS reduction, and microbiota restoration in treatment strategies. For more effective management of male infertility, further exploration of the molecular links between infections, inflammation, and OS is needed [110].

## 11. Conclusions

Microbial infections, inflammation, and OS contribute to male infertility, a multifaceted condition that damages reproductive tissue and impairs sperm function. These factors interact to create a vicious cycle, disrupting spermatogenesis, damaging sperm DNA, and altering the reproductive microenvironment, which exacerbates infertility. In addition to preventing microbiota dysbiosis, maintaining male reproductive health requires early detection of infections and timely antibiotic treatment. To reduce inflammation and OS, it is important to adopt healthy lifestyle choices, including a balanced diet, exercising frequently, and limiting exposure to environmental contaminants. Improving male fertility and reproductive health will require a multifaceted strategy that includes lifestyle changes, OS reduction, infection control, and microbiome restoration.

## Figures and Tables

**Table 1:** A summary of key OS indicators and their clinical significance.

Biomarker	Measurement Method	Function	Clinical Relevance
Malondialdehyde (MDA)	Seminal plasma analysis	Lipid peroxidation and membrane damage	Increased MDA indicates oxidative damage to sperm membranes, impairing motility and membrane integrity.
Glutathione (GSH)	Seminal plasma or sperm cell analysis	Antioxidant capacity	Low levels of GSH suggest insufficient antioxidant defenses, leading to increased vulnerability to ROS-induced damage.
Reactive Oxygen Species (ROS)	Chemiluminescence or fluorescence-based assays	Overall OS in semen	Elevated ROS levels correlate with conditions like asthenozoospermia and teratozoospermia.
DNA Fragmentation	Sperm DNA fragmentation assay (TUNEL, SCSA, etc.)	DNA damage in sperm	Higher DNA fragmentation is associated with poor fertility outcomes, including lower fertilization rates and early pregnancy loss.
Chromatin Condensation	Chromatin condensation assay (e.g., chromomycin A3)	Sperm nuclear structure	Impaired chromatin condensation indicates potential DNA damage and reduced sperm viability.

## Data Availability

No new data were created or analyzed in this study. Data sharing is not applicable to this article.
